# Development of a lateral flow device for rapid simultaneous multiple detections of some common bacterial causes of bovine mastitis

**DOI:** 10.5455/javar.2023.j681

**Published:** 2023-06-30

**Authors:** Rafik Hamed Sayed, Rafik Twfik Soliman, Shaimaa Abdelall Elsaady

**Affiliations:** 1 CLEVB, Agriculture Research Center, Cairo, Egypt; 2 Department of Microbiology, Faculty of Veterinary Medicine, Cairo University, Cairo, Egypt

**Keywords:** Bovine mastitis, bacterial pathogens, lateral flow test

## Abstract

**Objective:**

This work was conducted for the development of a 5-combi lateral flow immunochromatographic kit (LFK) for rapid and simultaneous identification of the common bacterial causes of bovine mastitis. The following pathogens are the identification targets of this kit: *Escherichia coli*,* Staphylococcus aureus*, *Klebsiella pneumoniae*,* Streptococcus agalactiae*, and* Streptococcus pyogenes* in milk samples from suspected bovine mastitis cases. The conventional microbiological identification of these agents is not only time-consuming and requires a fully equipped laboratory but also requires experienced personnel.

**Materials and Methods:**

Rabbit polyclonal antibodies (PAbs) specific to the antigenic components of the selected pathogens were prepared, and the pathogen-specific IgG was separated, purified, and conjugated with nanogold that was laid on the conjugate pad. Guinea pig PAbs specific to the microbial antigens of the selected pathogens were prepared, and their IgG content was separated, purified, and used as a capture antibody in the test (T) line on the nitrocellulose (NC) strips. Goat anti-rabbit IgG antibodies were used to capture the rabbit antibodies in the control (C) line of NC strips. The kit was held in a device comprising five strip-holding channels for the above-mentioned five bacterial species antigens. The developed LFK was evaluated, and its sensitivity and specificity were determined.

**Results:**

The developed kits were applied for the examination of bovine milk samples from suspected mastitis cases, and the average sensitivity, specificity, and accuracy of 5-combi LFK for the detection of the five selected bacterial species compared to bacteriological examination (gold standard test) were 93.90%, 80.83%, and 90.53%, respectively. The minimal microbial count that gave positive results using the developed LFK was 10^3 ^colony forming unit/ml. Treatment of the milk samples with an application buffer and its pre-incubation in trypticase soy broth for 6 h at 37°C before testing significantly increased the sensitivity of the prepared LFK. The developed kit proved simple and convenient, and the results could be obtained in less than 10 min.

## Introduction

Infection of the udder and the development of bovine mastitis are among the important infectious diseases affecting the dairy industry [1,2]. Bovine mastitis is a multifactorial disease and is one of the most difficult infections to control. Several bacterial species are implicated as causes of bovine mastitis. The most recorded microbial species include *Staphylococcus aureus*,* Streptococcus pyogenes*,* Streptococcus agalactiae*,* Pseudomonas aeruginosa*,* Klebsiella pneumoniae*,* Escherichia coli*, and *Candida* [3,4].

For the diagnosis of bovine mastitis cases, more attention has been given to the indirect test, which depends on the cellular reaction between reagents and certain protein factors in mastitic milk. These indirect tests include the somatic cell count (SCC) [5] and the California mastitis test (CMT) [6]. The bacteriological isolation of the causative microorganisms is the most accurate approach; however, it is expensive and time-consuming, requiring a fully equipped laboratory and experienced personnel. The polymerase chain reaction (PCR), on the other hand, is a specific and sensitive test for the detection of clinical and subclinical mastitis and can determine the pathogen in milk samples at the species level in a long time period of 2–4 h [7]. However, it is expensive and needs special materials and equipment for application.

The lateral flow immunochromatographic kit (LFK) is a simple, rapid test that can be carried out in the field. Now the LFK technology is widely used for the determination of bacterial pathogens in clinical samples of different diseases, such as *S. aureus* in clinical samples [8], *S. aureus* enterotoxin A in milk [9], *Vibrio cholera* [10], *Vibrio harveyi* [11], *Yersinia pestis* in humans [12], Leptospira in urine [13], *Helicobacter pylori* in stool [14], *Streptococcus suis* serotype 2 [15], *Salmonella enterica* subsp. Enterica serovar Typhimurium (*S. typhimurium*) [16,17], *Candida albicans* [18], and COVID-19 coronavirus [19].

The present work was designed to make a 5-combi LFK for simultaneous multiple and fast identification in milk samples of any of the following bacterial causes of bovine mastitis, namely, *S. aureus*,* E. coli*,* K. pneumoniae*,* S. agalactiae*, and* S. pyogenes.*

## Materials and Methods

### Institutional review board statement

The Institutional Animal Care and Use Committee at the Central Laboratory for Evaluation of Veterinary Biologics approved the research manuscript, which has been reviewed under our research authority and fulfills bioethical standards.

### Bacterial strains used 

The following bacterial species were used in the study: *E. coli* (ATCC#25922), *S. aureus* (ATCC#25923), *K. pneumoniae* (ATCC#700603), *S. agalactiae *(ATCC# 12386), and* S. pyogenes *(ATCC# 19615). The used strains were kindly supplied by the Laboratory for Control and Evaluation of Biological Preparations, Abbasia, Egypt. The strains were fully identified using bacteriological and molecular procedures.

### Preparation of specific bacterial antigens 

Cultures of each of the above-mentioned five bacterial species were harvested into chilled centrifuge tubes containing 4 ml of sterile phosphate-buffered saline (PBS) and washed four times using PBS by centrifugation at 3,000 × gm for 15 min. The harvested cells of each bacterial species were disrupted by sonication, and the whole antigen preparation of each of the five bacterial species was adjusted to 2 mg/ml using sterile PBS [20].

### Preparation of polyclonal antibodies (PAbs) against the specific antigens of each of the five selected bacterial species

Preparation of antigen-specific PAbs in guinea pigs against each of the five selected bacterial species according to Gulbenkian et al. [21]. The soluble bacterial antigen from each of the five bacterial species was emulsified with an equal volume of complete Freund’s adjuvant and used for the first immunization of guinea pigs (200 μg antigen/dose injected subcutaneously). The booster doses for each of the five bacterial species were prepared by mixing the whole cell antigen of each species with an equal volume of incomplete Freund’s adjuvant. Four successive booster doses (100 μg antigen/dose) were S/C inoculated at 2-week intervals in the immunized guinea pig. Then, 1 week after the last injection, the serum collection was checked for the presence of specific antibodies against bacterial antigens.

### Preparation of PAbs against each of the five selected bacterial species in rabbits 

Concentration and purification of microbial-specific rabbit and guinea pig IgG using the caprylic acid method according to Rafik et al*.* [19]: The species-specific IgG against each of the selected bacterial species was separated and purified from rabbit serum as follows: 50 ml of 0.06 M sodium acetate buffer (pH 4.6) was mixed with 25 ml of serum samples in a flask with a magnetic stirrer. After centrifugation of serum at 10,000 × gm for 20–30 min, the pellet was discarded. To the collected supernatant, 2.05 ml of caprylic acids were added slowly dropwise for 30 min at room temperature and then centrifuged at 10,000 × gm for 20 min. The pellet was discarded. The supernatant was dialyzed against PBS buffer at 4°C overnight using three buffer changes. The same procedure was applied for the separation and purification of bacterial-specific IgG from guinea pig serum; however, the amount of caprylic acid used was 2 ml/25 ml of serum [22].

### Removal of cross-reactivity in the prepared antisera by adsorption procedure

Each of the five prepared bacterial species-specific antisera was absorbed by the other four microbial antigens to remove any cross-reactive antibodies. The antiserum antigen mixture was incubated at 37°C for 1 h. This was followed by centrifugation of the mixture for 10 min at 1,000 × gm, and the serum was collected and stored at 4°C [23].

### Preparation of colloidal gold nanoparticles

#### Conjugation of each bacterial species-specific rabbit IgG with colloidal gold

A colloid gold solution was adjusted to pH 8.5 using 0.02 M K_2_CO_3_. 100 ul of rabbit IgG antibody (2 mg/0.1 ml of 0.05% NaCl buffer) were added slowly to 10 ml of the adjusted colloid gold solution while stirring gently. The mixture was mixed for 10 min and then blocked with a final concentration of 1% (m/v) of polyethylene glycol (PEG—20,000 kDa) [16,24].

### Preparation of the immunochromatographic lateral flow kit

#### Sample pad (Ahlstrom)

The material is composed of glass fiber, and sample pads were soaked in a PBS solution with a pH value of 7.2. The solution contained 0.3% Tween-20 and 0.5% (w/v) triton ×100 and was subsequently air-dried at 37°C. The material was then stored in a dry environment at room temperature until required for use [15].

#### The conjugate pad (Ahlstrom)

A glass fiber conjugate pad was prepared by treating it with 0.1% Tween-20 and drying it at 60°C. Next, it was saturated with 150 μl of the colloidal gold probe and dried at 37°C for 1 h. Finally, the conjugate pad was stored under dry conditions at 4°C until use.

#### Nitrocellulose (NC) membrane (BIODOT-XYZ-3)

**Figure 1.**
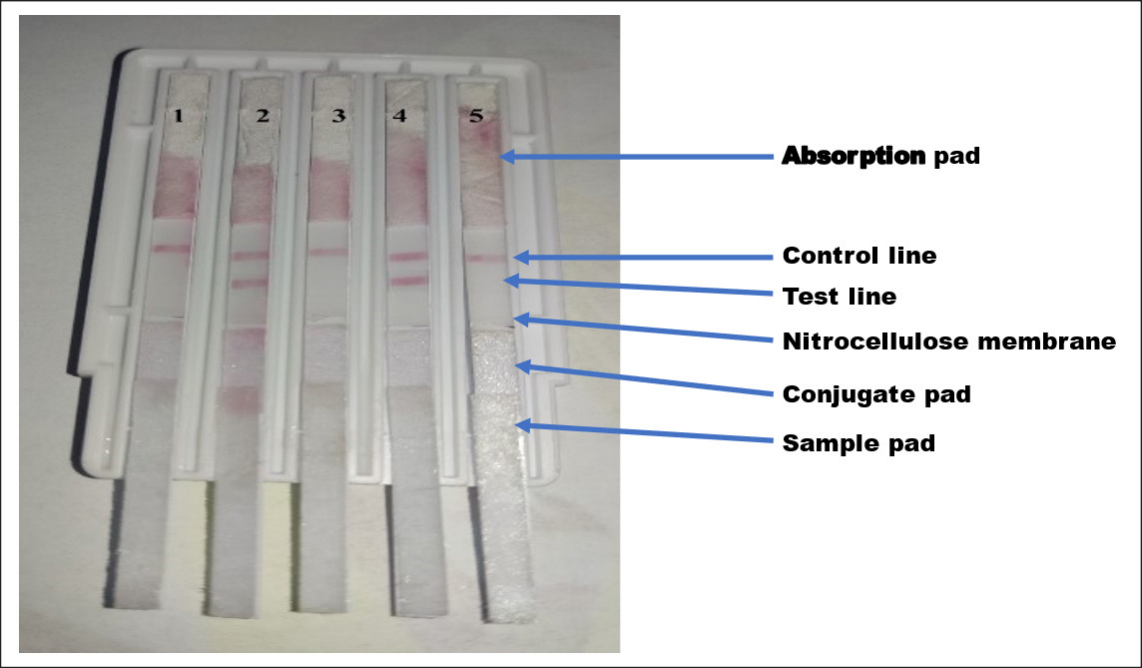
The 5-combi channel cassette contains five strips: (1)* S. aureus*, (2)* E. coli*, (3) *K. pneumoniae,* (4)* S. agalactiae*, and (5)* S. pyogenes. *The tested milk sample was a mixed infection with *E. coli* and *S. agalactiae*.

Two lines were dispensed on the NC membrane (25 × 300 mm): The bacterial species-specific guinea pig IgG (1.5 mg/0.1 ml) was dispensed around the bottom of the test (T) line (1 μl per 1 cm line). The control line (C) was created using goat anti-rabbit IgG at 1 mg/ml concentration and was dispensed at the top of the membrane. Each line was 1 μl per 1 cm and was kept at a distance of 5 mm from each other. The membrane was then covered with a top laminate and cut into strips of 0.5 cm width with an automated cutter (Guillotino Cutter GCI1800), The strips of five microorganisms were loaded on the 5-combi channel cassette as shown in Figure 1.

### Determination of the specificity of the developed bovine mastitis diagnostic LFK

Pure cultures of five selected bacterial pathogens, namely, *S. aureus*,* E. coli*,* K. pneumoniae*, *S. agalactiae*, and* S. pyogenes* were grown on trypticase soy agar for 3 h. The harvested culture was suspended in the application buffer and tested with the developed LFK.

### Sensitivity testing of the developed bovine mastitis diagnostic LFK

Each of the five bacterial species was tenfold serially diluted (10^1^–10^8^) with the application buffer, and the bacterial suspension at each dilution was tested by the developed LFK.

### Determination of the effect of pre-enrichment of the tested bacterial samples on the sensitivity of the developed LFK

From each of the five bacterial species, different dilutions from 10^1^ to 10^6 ^colony forming unit (CFU)/ml were prepared in trypticase soy broth (TSB) and incubated at 37°C for different incubation times ½, 1, 2, 3, and 6 h, then suddenly killed by heating at 60°C for 30 min and testing with the developed LFK.

### Evaluation of the diagnostic efficacy of the prepared LFK in rapid simultaneous detection of any of these five selected microbial pathogens causing bovine mastitis

250 bovine milk samples collected from clinically suspected mastitis were examined both bacteriologically (gold standard test) and by the developed LFK. The efficacy of the developed LFK was determined through the evaluation of its sensitivity, specificity, and accuracy as compared with the bacteriological isolation.

### Milk samples

A total number of 250 milk samples were collected aseptically from dairy cattle. The samples were collected from cases either with atrophied quarters, dried, or suffering from severe mastitis according to the procedures of Radostits et al. [25]. From each quarter, 15–20 ml milk samples were collected in a clean, sterile, labeled screw-capped bottle. Three milk samples were collected and kept in an ice container until delivered to the laboratory. One of the three samples was examined for SCC (this sample was kept in formalin 10% if it was not examined for the SCC on the same day). The second sample was subjected to a lateral flow test according to the results of CMT and SCC. The third sample was subjected to bacteriological examination after being pre-incubated for 24 h.

### Determination of the effect of sample preparation on the sensitivity and specificity of the developed lateral flow diagnostic kit

The tested milk samples were examined untreated and after being treated with application buffer to determine the best pretesting preparation protocol. The milk samples were tested in the following forms:

A. Untreated milk samples.

B. Milk sample treated with application buffer.

C. Untreated Milk whey: The milk whey was prepared from the collected milk samples, according to Fetrow [26].

D. Milk whey treated with application buffer.

### Treatment of tested milk samples with application buffer

An application buffer prepared according to Sithigorngul [11] and composed of 336 mM NaCl 30 mM Tris, 9 mM EDTA, and 1% tween 80 (pH 9.3) was used to treat the milk samples. Each milk sample was mixed with the application buffer and incubated for 15 min just before being tested with the LFK.

### Statistical analysis and evaluation of diagnostic kits (test validity)

The developed LFK was applied to detect any of the five bacterial pathogens, namely, *S. aureus*,* E. coli*, *K. pneumoniae*, *S. agalactiae*, and* S. pyogenes *in milk samples from suspected bovine mastitis cases. The obtained results were compared with the results of direct bacteriological examination (gold standard test) of the same samples. The sensitivity, specificity, and accuracy of the developed LFK were determined using the Statistix^®^ (1996) package.

## Results

### Results of examination of the bovine milk samples using the conventional procedures, namely, CMT and SCC 

The collected milk samples were examined for the presence of mastitis using CMT and SCC (Table 1). Using CMT, the percentage of apparently healthy, subclinical mastitis, and clinical mastitis milk samples were 51 samples (20.4%), 120 samples (48%), and 79 samples (31.6%), respectively.

The examination of 120 milk samples from suspected subclinical mastitis cases using the SCC test showed the following results: 32 samples were in the range of <10^5^ SCC/ml, 60 samples were in the range of >10^5^ − 3 × 10^5 ^SSC/ml, and 28 samples in the range of >3 × 10^5 ^− 5 × 10^5^ SCC/ml.

On the other hand, an examination of 79 milk samples from suspected clinical mastitic cases using the SCC test revealed the following: 51 cases showed SCC in the range of >5 × 10^5^ − 10^6^ SCC/ml, and 28 cases showed SCC in the range of >10^6^ SCC/ml.

**Table 1.** Results of examination of bovine milk samples using CMT and SCC.

The test interpretation	Milk samples			
	CMT		SCC/ml	
	No. of cows	%	Ranges	No. of cows
Healthy	51	20.4	<10^5^	51
Subclinical mastitis	120	48.0	<10^5^	32
			>1 × 10^5^ − 3 × 10^5^	60
			>3 × 10^5 ^− 5 × 10^5^	28
Clinical mastitis	79	31.6	>5 × 10^5 ^− 1 × 10^6^	51
			>10^6^	28
Total	250			250

### Results of bacteriological examination of the CMT-positive milk samples 

Bacteriological examination of the 199 positive CMT milk samples revealed that 190 of the tested samples were bacteriologically positive (94.95%), while 9 samples (5.03%) were negative (Table 2).

The number of cases associated with single infection, mixed infection, and bacteriological free samples were 109 (54.77%), 81 (40.7%), and 9 (4.5%), respectively. In milk samples with a single infection, the identified bacterial agents were as follows;* E. coli *(13.8%), *S. aureus* (21.2%), CNS (7%), *S. agalactiae* (17.5%), *S. pyogenes *(18.5%), *K. pneumoniae *(16.6 %), *Salmonella *spp. (0.9 %), *Proteus *sp. (0.9%), *P. aeruginosa* (1.8%), and *C. albicans *(0.9%).

In the case of the mixed infection, the recovered bacterial agents were *S. aureus *plus *E. coli *(20.9%),* E. coli *plus* K. pneumoniae *(14.8%),* S. aureus *plus* K. pneumoniae *(11.1%), CNS plus *E. coli *(7.4%),* S. aureus* plus* S. agalactiae* and* E. coli* (18.5%),* S. aureus *plus* E. coli *and* K. pneumoniae *(11.1%),* S. agalactiae *plus* E. coli* (8.6%), *S. pyogenes* plus* E. coli *(4.9%), CNS plus* K. pneumoniae *(1.2%) and *Proteus *spp. plus* S. aureus *(1.2%).

### Specificity of the developed lateral flow kits

The prepared LFK gave positive results when tested against the corresponding bacterial pathogen and gave clear negative results against the other four bacterial species and different other microbial species, including* C. albicans *and* P. aeruginosa*.

### Sensitivity of the developed lateral flow kit (LFK)

**Figure 2.**
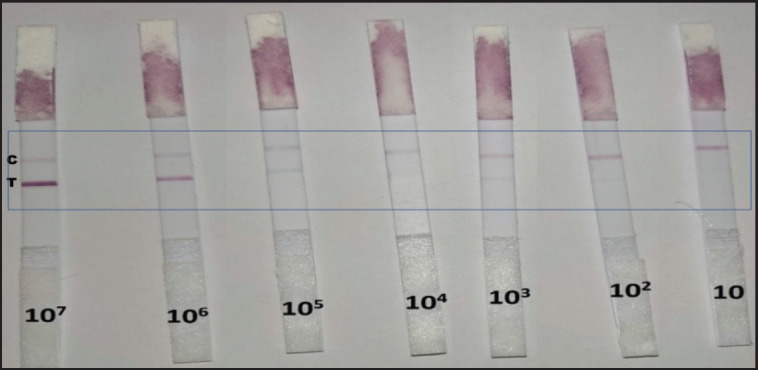
Sensitivity of the prepared LFK in detecting *S. aureus* in clinical samples.

The lowest concentrations of the bacterial cultures (*E. coli*,* S. aureus*,* K. pneumoniae*,* S. agalactiae*, and *S. pyogenes*) that gave positive results were as follows (Table 3 and Fig. 2):

**Table 2.** Incidence of bacterial species recovered in the bacteriological examination of CMT-positive milk samples.

Single bacterial infection				Mixed bacterial infection			
No. of milk samples	Bacterial species	No. of isolates	%	No. of milk samples	Bacterial species	No. of isolates	%
109	*E. coli*	15	13.76%		*S. aureus *and* E. coli*	17	20.99%
	*S. aureus*	23	21.10%		*S. aureus*,* S. agalactiae *and* E. coli*	15	18.51%
	Coagulase-negative* Staphylococci *(CNS)	9	8.25%		*S. aureus*,* E. coli *and* K. pneumoniae*	9	11.11%
	*S. agalactiae*	19	17.43%		*E. coli *and* K. pneumoniae*	12	14.82%
	*S. pyogenes*	20	18.34%		*S. aureus *and* K. pneumoniae*	9	11.11%
	*K. pneumoniae*	18	16.51%		CNS and* E. coli*	6	7.41%
	*Salmonella *spp.	1	0.92%		*S. agalactiae *and* E. coli*	7	8.64%
	*Proteus *spp.	1	0.92%		*S. pyogenes *and* E. coli*	4	4.94%
	*P. aeruginosa*	2	1.84%		CNS and* K. pneumoniae*	1	1.23%
	*C. albicans*	1	0.92%		*Proteus *spp. and* S. aureus*	1	1.23%
Total		109		Total		81	

**Table 3.** Sensitivity of the prepared lateral flow kits in the detection of *E. coli*, *S. aureus*, and *K. pneumoniae *causing bovine mastitis.

Count*	0	10^1^	10^2^	10^3^	10^4^	10^5^	10^6^	10^7^	10^8^
*E. coli*	−	−	−	−/+	+	++	+++	+++	+++
*S. aureus*	−	−	−	−/+	+	++	+++	+++	+++
*K. pneumoniae*	−	−	−	−/+	+	++	+++	+++	+++
*S. agalactiae*	−	−	−	−/+	+	++	+++	+++	+++
*S. pyogenes*	−	−	−	−/+	+	++	+++	+++	+++

**Figure 3.**
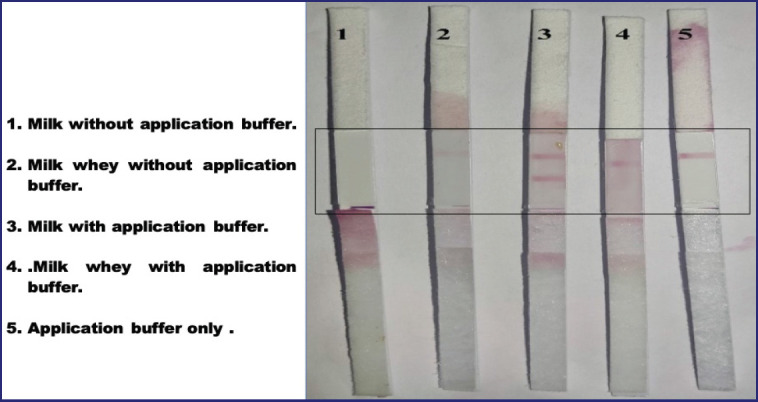
Effect of methods of milk sample treatment on the performance of the LFK for detection of *E. coli*.

– 10^3^ CFU/ml = color degree was suspected (−/+).

– 10^4^ CFU/ml = Weak positive (+).

– 10^5 ^CFU/ml = Positive (++).

– 10^6^ CFU/ml = Strong positive (+++).

### Effect of the treatment method of the milk samples before testing on the sensitivity of the developed LFK

The examined milk samples were tested in different forms, including untreated fresh milk samples, milk samples treated with application buffer, untreated milk whey, and milk whey treated with application buffer. The differently-treated milk samples were examined with the developed LFK, and the best results were recorded in milk samples treated with the application buffer (Fig. 3).

The 199 milk samples that were positive in the bacteriological examination (10^9^ with single infection and 81 with mixed infections) were tested with the developed LFK. As shown in Table 4, the determined sensitivity, specificity, and accuracy of the developed LFK compared to bacteriological examination was 95.29%, 80.00%, and 95.51%, respectively, for *E. coli*; 97.50%, 78.57%, and 94.68%, respectively, for* S. aureus*; 93.61%, 83.33% and 92.45%, respectively, for* K. pneumoniae*; 89.19%, 80.00% and 87.23%, respectively for *S. agalactiae*; and 83.33%, 80.00%, and 82.76%, respectively for *S. pyogenes*. The average sensitivity of the developed 5-combi LFK for identifying the five selected bacterial pathogens was 90.2%, 75.5%, and 88.5%, respectively.

**Table 4.** Sensitivity, specificity, and accuracy of 5-combi LFK in detecting the five bacterial species causing bovine mastitis compared to bacteriological examination.

			Bacteriological examination			Sensitivity	Specificity	Accuracy
			(+) ve	Total	(−) ve			
**LFK**	*E.coli*	(+) ve	TP 77	FP 1	78	95.29%	80.00%	95.51%
		(−) ve	FN 4	TN 3	7			
		Total	81	4	85			
	*S. aureus *and (CNS)	(+) ve	TP 76	FP 3	79	97.50%	78.57%	94.68%
		(−) ve	FN 2	TN 8	10			
		Total	78	11	89			
	*K. pneumoniae*	(+) ve	TP 40	FP 1	41	93.61%	83.33%	92.45%
		(−) ve	FN 4	TN 4	8			
		Total	44	5	49			
	*S. agalactiae*	(+) ve	TP 29	FP 2	31	89.19%	80.00%	87.23%
		(−) ve	FN 4	TN 6	10			
		Total	33	8	41			
	*S. pyogenes*	(+) ve	TP 16	FP 1	17	83.33%	80.00%	82.76%
		(−) ve	FN 4	TN 3	7			
		Total	20	4	24			
	Average					93.90%	80.83%	90.53%

TP = True positive; TN = True negative; FP = False positive; FN = False negative.

Sensitivity = TP/ (TP + FN), Specificity = TN/ (TN + FP), and Accuracy = (TN + TP)/(TN+TP+FN+FP).

Effect of the incubation time in TBS medium of tested microbial strains on the sensitivity of prepared lateral flow kits: The minimum incubation time needed to achieve the highest sensitivity of the developed bovine mastitis lateral flow diagnostic kits were 6 h incubation time at 37°C, which gave the best result and high sensitivity

## Discussion

Laboratory diagnosis of bovine mastitis through bacteriological isolation and identification of the causative microorganisms is the gold master test. However, it is expensive, time-consuming, and requires a fully equipped laboratory and experienced staff [27] Moreover, the molecular diagnosis of bovine mastitis using PCR is sensitive and specific for the diagnosis of clinical and subclinical mastitis and could be used in the detection of pathogens in milk samples at the species level in few hours [28,29]. However, again, it is expensive, not suitable as a field test, and like the bacteriological examination, requires a specially equipped laboratory.

The ideal diagnostic tool for bovine mastitis should be able to detect the causative agent in the shortest possible time. A rapid diagnostic tool is fundamental for the management of udder health, and the earlier the disease is identified, the less will be the damage. Moreover, it should be simple, sensitive, specific, economical, and suitable as a field or laboratory test that can be applied to a large scale of animals. Such a diagnostic approach, when developed, will facilitate the application of the correct treatment method at the proper time and reduce the complication of mastitis. The criteria of the lateral flow immunoassay as a simple, rapid test that can be applied in the field nominate it as an ideal approach for rapid identification of the microbial causes of bovine mastitis. In the present work, a lateral flow kit (LFK) has been developed for rapid, multiple detection of any of the five common causes of bovine mastitis, namely, *E. coli*,* S. aureus*,* K. pneumoniae*,* S. agalactiae *and* S. pyogenes *in milk samples from suspected cases of bovine mastitis. The sensitivity, specificity, and accuracy of the developed kit were determined using the bacteriological examination of the milk samples as a master gold test.

The sensitivity of the prepared LFK was achieved by determining the minimum bacterial count/ml that can be detected by the developed kit, which reached 10^3^/ml for the five studied bacterial species (Fig. 2). These results are comparable to those of Humar et al. [30], who recorded a sensitivity of 100 CFU/100 μl of* S. enterica* serovar Typhi using plate ELISA. Also, Blaskoza et al. [31] estimated a sensitivity of 10 CFU/25 μl of *Listeria monocytogenes *in dairy products using a lateral flow Device. On the other hand, Wiriyachaiporn et al. [32] showed that the sensitivity of the lateral flow immunochromatographic device for the detection of* S. aureus *from bronchoalveolar lavage samples was 10^6^ CFU/ml, while Jung et al. [33] reported a sensitivity of 10^5^ CFU/gm of lateral flow devices for *E. coli* O157: H7 in bovine feces. Such variation in the sensitivity of the lateral flow immunoassay can be attributed to several factors, the most important one is the sensitivity and affinity of the used antibodies. The increase in the sensitivity of our prepared kits is attributed to the use of two types of specific PAbs for each pathogen, the first one (primary antibodies) was the gold chloride conjugated pathogen-specific antibody prepared in rabbits and placed in the conjugate pad for catching the pathogen antigens present in the tested sample. The second antibody (secondary antibody), however, was prepared in different animal species, namely, guinea pigs, and was placed in the test line to catch the pathogen-specific antigen-antibody complex developed in the conjugate pad. The two types of antibodies prepared in different animal species might recognize different epitopes on the same antigen molecules, which stand behind the increasing sensitivity of the developed kit. A similar approach has been described by O’Keeff et al. [34]. 

To determine the sensitivity, specificity, and accuracy of the prepared kit for multiple detections of any of the five selected pathogens, the collected bovine milk samples were examined both by the developed kit and bacteriologically, and the results were compared. The determined sensitivity, specificity, and accuracy of the developed LFD compared to bacteriological examination was 95.29%, 80.00%, and 95.51%, respectively, for *E. coli*; 97.50%, 78.57%, and 94.68%, respectively, for* S. aureus*; 93.61%, 83.33%, and 92.45%, respectively, for *K. pneumoniae*. 89.19%, 80.00%, and 87.23%, respectively, for *S. agalactiae*; and 83.33%, 80.00% and 82.76%%, respectively for* S. pyogenes. *These results are comparable to those recorded by Fang et al. [35], who recorded a sensitivity and specificity of 100% and 99%, respectively, for detecting *Salmonella* spp. using LFK compared to bacteriological examination. Also, Bautista et al. [36] reported a sensitivity rate of 12.3% and a specificity of 100% of the lateral flow strips developed for detecting *S. typhimurium* in chickens. The variation in the sensitivity and specificity of the LFK can be attributed to many factors, the most important of which is the quality and specificity of the prepared pathogen-specific antibodies.

The effect of pre-incubation of the tested samples in a TSB medium on the sensitivity of the developed kits was determined. The minimal time required for the pre-enrichment to get the suspected positive reading with the 10 CFU/ml sample was 6 h in TSB, as the positive results were observed with the tested samples. Humar et al. [30] proved that pre-incubation of tested samples for 4 h in brain heart infusion broth could increase the LFK sensitivity for detecting *Salmonella* in water at least 10 times. Also, Seo et al. [37] reported a sensitivity of 100% for *S. enteriditis* in raw egg pools inoculated with 10 *S. enteriditis* cells per ml of egg and incubated in buffered peptone water or tetrathionate brilliant green broth for 24 h at 37°C. Sithigorngul et al. [11] recorded an increase in the sensitivity of LFK strips for detection of *V. harveyi* to 1–10 CFU/ml in the test sample through pre-incubation in TSB for 6 h before application of the strip. Such sensitivity is comparable to that of PCR. The pretesting treatment of the milk samples of application buffer associated with its pre-incubation in TSB for 6 h at 37°C significantly increased the sensitivity of the developed LFK in detecting any of the five selected pathogens.

## Conclusion

In the present work, an LFK was developed to diagnose bovine mastitis as a field test. Compared with the bacteriological examination, the developed kit proved sensitive, specific, and accurate in the diagnosis of bovine mastitis caused by the following bacterial pathogens;* E. coli*,* S. aureus*,* K. pneumoniae*,* S. agalactiae*, and* S. pyogenes*. The developed LFK was not only very rapid (5–10 min) but also simple, convenient, had a long shelf time, and can be used by untrained personnel at the dairy farm site without requiring additional equipment.

## List of abbreviations

LFK, lateral flow immunochromatographic kit; PAbs, polyclonal antibodies; NC, nitrocellulose; CFU, colony forming units; TSB, Trypticase soy broth; SCC, Somatic cell count; CMT, California mastitis test; PCR, Polymerase chain reaction.
